# Individualized prevention against hypertension based on Traditional Chinese Medicine Constitution Theory: A large community-based retrospective, STROBE-compliant study among Chinese population

**DOI:** 10.1097/MD.0000000000008513

**Published:** 2017-11-17

**Authors:** Ying Li, Xiao-Hui Li, Xin Huang, Lu Yin, Cheng-Xian Guo, Chang Liu, Yong-Mei He, Xing Liu, Hong Yuan

**Affiliations:** a Department of Health Management, The Third Xiangya Hospital; b Health Management Research Center, Central South University; c Department of Pharmacology, School of Pharmaceutical Sciences, Central South University; d Preventive Medicine, Medical School of Hunan Normal University, Changsha; e State Key Laboratory of Cardiovascular Disease, Fuwai Hospital, National Center for Cardiovascular Disease, Peking Union Medical College & Chinese Academy of Medical Sciences, Beijing; f Center of Clinical Pharmacology, The Third Xiangya Hospital, Central South University, Changsha; g Second Department of Geriatric Medicine, Aerospace Center Hospital, Beijing, P. R. China.

**Keywords:** Chinese residents, hypertension, traditional Chinese Medicine Constitution

## Abstract

Traditional Chinese Medicine Constitution (TCMC) theory states that individuals with a biased TCMC are more likely to suffer from specific diseases. However, little is known regarding the influence of TCMC on susceptibility to hypertension. The aim of this study is to examine the possible relationship between TCMC and hypertension. Retrospective evaluation and observation were performed using the STROBE guidelines checklist. A large community-based cross-sectional study was conducted between 2009 and 2013 in Changsha, China. TCMC was assessed using a questionnaire that included 68 items. TCMC distributions and the associations of different TCMCs with hypertension risk were analyzed. In total, 144,439 subjects underwent evaluations of TCMC and blood pressure (BP). There were significant differences in the hypertension prevalence among the various TCMC groups (*P* < .01). An adjusted logistic regression model indicated that those with phlegm wetness, yin deficiency, blood stasis, or qi deficiency were more likely to have hypertension. Analysis of the clinical characteristics related to TCMC indicated that different TCMCs corresponded to different hypertension classifications using Western medicine criteria; for example, phlegm wetness with hypertension was similar to obesity-related hypertension. Our results suggest that phlegm wetness, yin deficiency, blood stasis, and qi deficiency have different effects on the prevalence of hypertension. More attention should be paid to TCMCs associated with susceptibility to hypertension, and corresponding preventive and therapeutic treatments should be developed according to different TCMCs.

## Abstract 2

Supplemental Digital Content is available in the text

## 1 Introduction

Hypertension is a major risk factor for cardiovascular disease and related death.^[1]^ The prevalence of hypertension is 27.2% in the adult Chinese population aged 35 to 74 years^[2]^; in Europe, United States, and Canada, this prevalence is 44.2%, 27.8%, and 27.4%, respectively.^[3]^

In the Western world, human constitutions were described as early as in the writings of *Hippocrates of Cos*. Four types of constitution, namely, blood, phlegm, yellow bile, and black bile, were defined. Traditional Chinese Medicine Constitution (TCMC, called *Tizhi* in Chinese) was first mentioned in *Huang di*'s Canon of Medicine. TCMC is defined as an integrated, metastable, and inherent endowment based on innate and acquired traits of physical appearance, physiological function, and psychological condition.^[4]^ As a key evaluation standard for the Chinese population, TCMC theory has been widely applied for 2000 years to stratify individuals’ health statuses as subhealthy, subdisease, or pre-disease. Indeed, TCMC theory lays the foundation for health promotion and disease prevention in China.^[5]^

To improve nationwide health management and deliver better-personalized medicine, the Chinese government created a project to establish a normative TCMC system based on classified questionnaires administered by Chinese Mainland experts. The reproducibility, reliability, and validity of the questionnaire were assessed^[6]^ and then published by the China Association of Chinese Medicine in 2009 (Table S1–2).^[7]^ TCMCs are classified as either normal (N, also called *pinghe*) or biased (also called *Pianpo*) and can be further divided into 8 subtypes: qi deficiency (QDF), yang deficiency (PD), yin deficiency (ND), phlegm wetness (PW), wetness heat (WH), blood stasis (BS), qi depressed (QDP), and inherited special constitution (ISC). According to the defining criteria, a person's TCMC may be complex. Questionnaires and criteria classifying TCMC have been widely used in community health and physical examinations in China since 2009.

Recently, an increasing number of studies have focused on the distribution and formation of TCMC in individuals living in Mainland China, Taiwan, and Hong Kong. Wang et al carried out the largest-scale investigation, evaluating 21,948 people in different geographical locations in China. The results showed that a large number of participants suffer from biased TCMCs, such as the QDF, WH, and PD constitutions. Moreover, environmental location, sex, age, marital status, occupation, and education have been demonstrated to affect TCMC.^[8]^ Similar results were found in other studies.^[9]^ Furthermore, according to Traditional Chinese Medicine philosophy, people with a biased constitution are susceptible to subhealthy states and certain diseases, especially chronic diseases. Although a small sample study indicated that the PW, ND, and QDF constitutions are the major influencing factors on hypertension^[10]^ and that patients with the WH or QDP constitution might have increased susceptibility to intracerebral hemorrhage,^[11]^ additional studies using extensive data (including physical and blood test results) are still necessary to further elucidate the clinical relevance of TCMC and its relationship to hypertension.

This study aimed to investigate the demographic, physical, and clinical characteristics associated with different constitutions, as well as the relationship between TCMC and hypertension, in adult Chinese residents in Yuelu District, Changsha City. The results can potentially expand TCMC theory and have great significance for improving precision medicine to combat hypertension.

## 2 Materials and methods

### 2.1 Study design and data sources

This large community-based cross-sectional study collected information from community health registry systems in Yuelu District, Changsha City. Retrospective evaluation and observation were performed using the STROBE guidelines checklist. Community hospitals offer basic health services and also work to build profiles of the permanent resident population with resident consent. Some services are free according to “The National Basic Public Health Service Specification 2009.” TCMC classification of residents is required for those aged >65 years and is recommended for all residents. An official scorer uploads resident's electronic profiles to the community health registry system. The information includes personal details, health-related habits, family history, basic physical examination, and blood test results. The quantity and quality of electronic profiles is the main test of the community hospital. We acquired available data from the Yuelu Health Bureau without sensitive personal information, such as name or personal identification. Random sampling was not used to control for potential sources of bias; instead, we collected all information available based on eligibility criteria.

### 2.2 Participants

**Figure 1:**
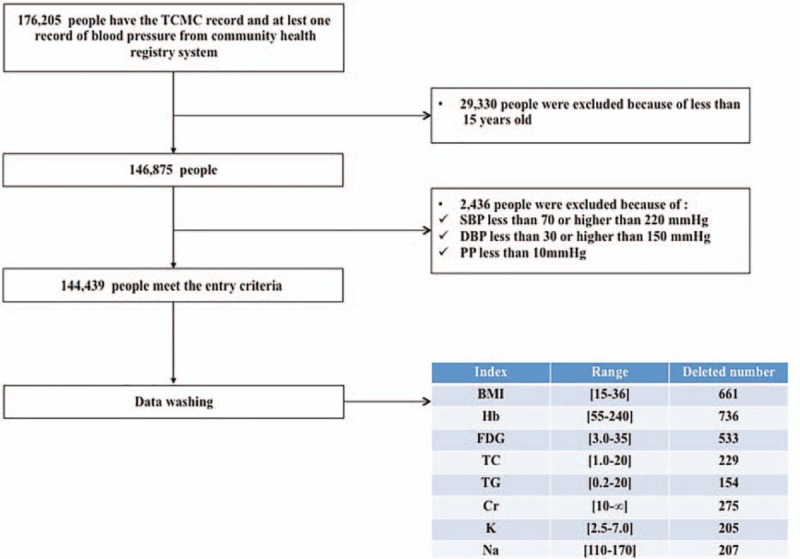
Flow diagram of study patient selection. The detailed inclusion protocol and data-cleaning criteria are presented. BMI = body mass index, Cr = creatinine, DBP = diastolic blood pressure, FBG = fasting blood glucose, Hb = hemoglobin, K = blood potassium, Na = blood sodium, PP, pulse pressure, SBP = systolic blood pressure, TC = total cholesterol, TG = triglyceride.

The participants were permanent residents older than 15 years with reasonable records of TCMC and BP. The participants were recruited from the community health registry system from January 1, 2009 to December 31, 2013, in Yuelu District, Changsha City, Hunan Province, China. If the participants had >1 record containing BP and blood test results, the most recent record was included in the study. Entry and data-cleaning criteria are shown in Figure 1.

### 2.3 Quality assurance of the clinical and laboratory data

Physicians in community hospitals performed the physical examinations and laboratory tests. Hypertension was defined as systolic blood pressure (SBP) ≥140 mmHg or diastolic blood pressure (DBP) ≥90 mmHg, or a history of hypertension, or taking antihypertensive drugs. Following the 2005 and 2010 Chinese Guidelines for the Prevention and Treatment of Hypertension, seated BP was measured at least twice using a mercury sphygmomanometer or electronic sphygmomanometer.

A fasting blood glucose (FBG) test was performed for free for older (>65 years’ old), hypertensive, and diabetic patients as well as for patients with serious mental illnesses. Extreme or irrational values for body mass index (BMI), FBG, hemoglobin (HB), total cholesterol (TC), triglyceride (TG), creatinine (Cr), blood sodium (Na), and potassium (K) were excluded (Fig. 1).

### 2.4 TCMC measurements

TCMC classifications were determined using questionnaires based on the TCMC classification and criteria issued by the China Association of Chinese Medicine (ZYYXH/T157–2009).^[7]^ The questionnaires consisted of 68 items on a 1 to 5 type response scale (from 1 [never happened] to 5 [always happens]) for 9 TCMC subtables, and each subtable had 7 or 8 items. The questionnaires and criteria are shown in Tables S1 and S2. Individuals answering “yes” for the N constitution produced same results to those answering “basically yes,” and individuals answering “yes” for the *Pianpo* constitution produced same results to those answering “possible yes” in statistical analysis. Either Traditional Chinese Medicine physicians or a special physician who had acquired professional training certification performed the diagnosis in the community hospitals.

### 2.5 Ethics statement

This study complied with the Declaration of Helsinki, and we guaranteed the participants that their data would be used only for scientific research. The institutional review board (IRB) of The Third Xiangya Hospital, Central South University (No. 2015-S164), approved the study. The IRB membership included community representatives and medicine and law experts. The residents provided written informed consent for the use of their health information by the research center.

### 2.6 Statistical analysis

All analyses were performed using SPSS (version 17.0) statistical software with 2-sided tests. Continuous variables are shown as means and 95% confidence intervals (CIs), and categorical variables are shown as numbers (n) and percentages (%). Differences across groups were compared by analysis of variance (ANOVA)/nonparametric tests and *χ*^2^ tests, as appropriate. Crude and adjusted odd ratios (ORs) and their 95% CIs were calculated using unconditional logistic regression models to evaluate the associations between hypertension risk and 5 types of TCMC, including N, QDF, ND, PW, and BS. Adjusted logistic regression models were adjusted for age, sex (female vs. male), BMI (<18.5, 25–27.99, 28–31.99, and ≥32 kg/m^2^, with 18.5–24.99 as a reference), smoking (continued smoking and quit smoking, with never smoked as a reference), alcohol intake (sometimes, usually, and every day, with never as a reference), and family history (yes vs. no).

## 3 Results

### 3.1 Sociodemographic and physical characteristics

Data from the 176,205 participants who completed the TCMC questionnaires were collected from the community health registry system in Yuelu District, Changsha City. We organized the data to ensure the validity and veracity of the results. Participants who were missing BP measurements or who had a SBP <70 or <220 mmHg, a DBP <30 or >150 mmHg, or a pulse pressure (PP) <10 mmHg were removed. Ultimately, 144,439 participants were included in the final analysis (Figure 1).

The characteristics of the study participants are shown in Tables 1 and 2. The average age was 46.37 ± 17.69, with distributions of 15 to 34.9 years, 35 to 65 years, and older than 65 years constituting 31.8%, 49.4%, and 18.8% of the participants, respectively. Males made up 46.3% of the participants. The average BMI was 22.29 ± 2.18 kg/m^2^. Smokers constituted 6.3% of the participants: 1.0% of the participants had quit smoking, 91.1% of the participants had never smoked, and 1.6% of the participants had missing data. The frequencies of never drinking, sometimes drinking, usually drinking, and drinking everyday were 84.8%, 12.4%, 0.5%, and 0.3%, respectively, whereas 1.9% of the participants had missing data. In total, 6.8%, 0.7%, and 14.1% of the participants had diabetes, stroke, and coronary heart disease, respectively.

**Table 1:**
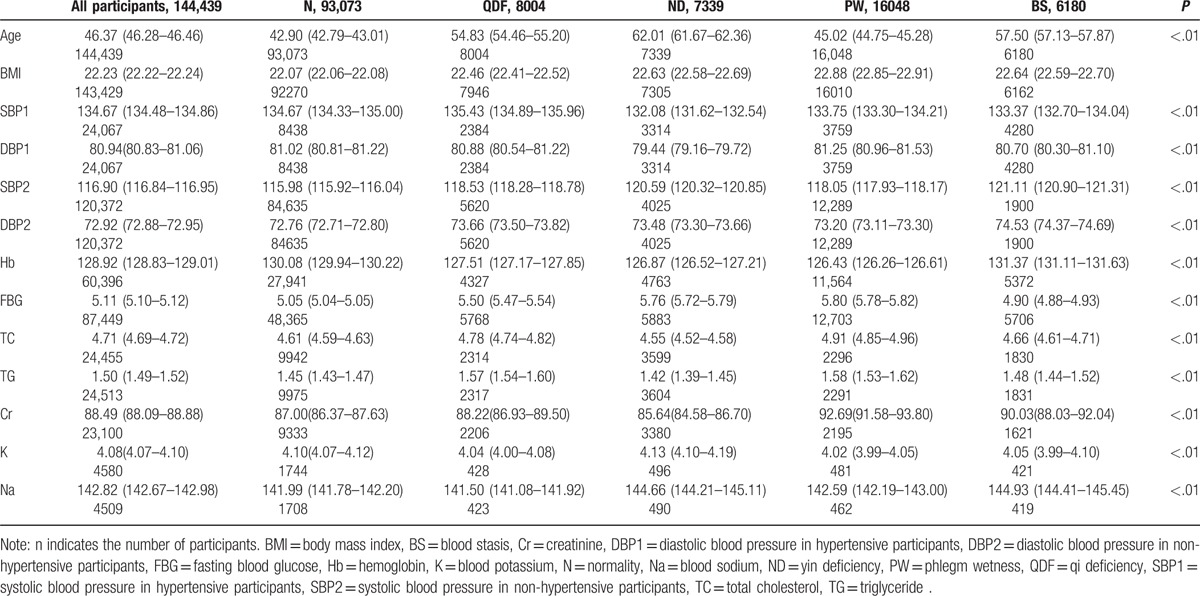
Comparison of the basic continuous variables corresponding to the characteristics of different TCMCs (mean values [95% confidence interval] [N]).

**Table 2:**
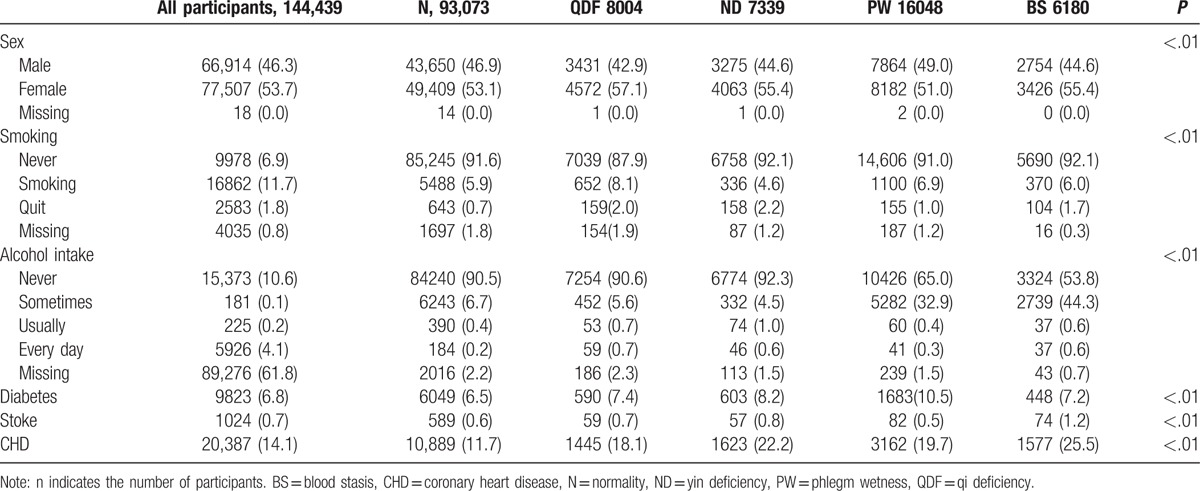
Comparison of the basic classified variables corresponding to the characteristics of different TCMCs (N [%]).

### 3.2 TCMC types

An individual can theoretically possess 1 to 9 TCMC types simultaneously. The majority (139,759) of participants had only 1 TCMC, whereas 4005, 547, and 128 participants had 2, 3, and >4 TCMCs, respectively. For the participants with a single TCMC, 94,496 (66.6%) participants presented the N constitution, whereas the frequencies of biased TCMCs were as follows: PW (11.5%), QDF (5.7%), ND (5.3%), BS (4.4%), PD (4.0%), QDP (1.3%), WH (1.0%), and ISC (0.2%).

### 3.3 Relationships between hypertension and TCMC types

The overall hypertension prevalence was 16.7% in all participants, and significant differences were apparent among individual TCMCs (*P* < .01). The prevalence of hypertension in the 9 TCMCs was as follows: ND (45.2%), BS (30.7%), QDF (29.8%), WH (28.1%), ISC (27.5%), PW (23.4%), PD (18.1%), QDP (11.0%), and N (9.1%). Based on the distribution of TCMCs and the hypertension prevalence in the various TCMCs, the PW, QDF, ND, and BS constitutions were chosen to analyze the relationship between hypertension and TCMC type. The blood pressure levels in hypertensive and nonhypertensive participants with the N, PW, QDF, ND, or BS constitution are shown in Table 1. The crude odds ratio (OR) for the relationship between TCMC and hypertension prevalence is presented in Table 3. Logistic regression showed that TCMC had an important impact on susceptibility to hypertension. Participants with PW (OR 2.56), ND (OR 1.98), BS (OR 1.55), or QDF (OR 1.39) TCMCs were more likely to experience hypertension after controlling for confounding factors, including age, sex, BMI, smoking, alcohol intake, and family history.

**Table 3:**
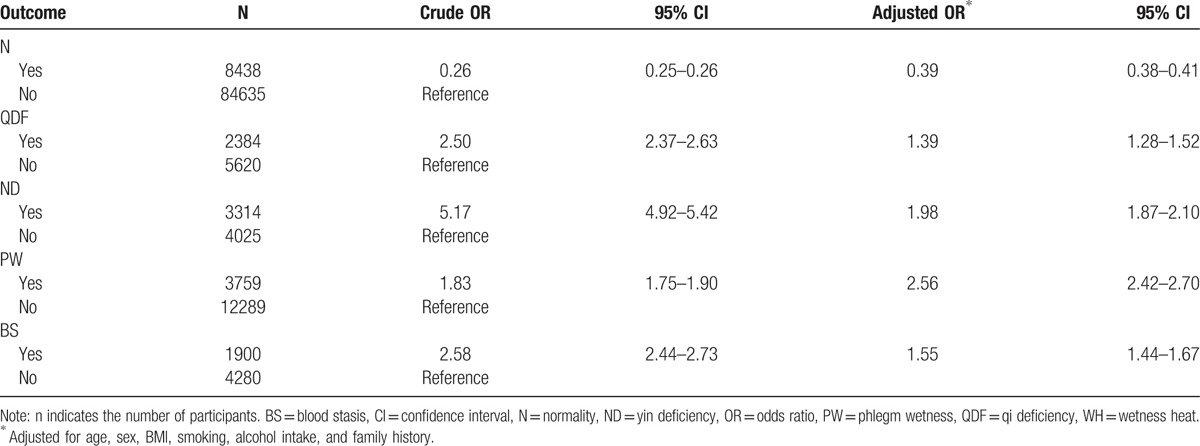
Relationship between TCMC and hypertension prevalence among the residents in Yuelu District.

### 3.4 Characteristics of the PW, QDF, ND, and BS TCMCs

Relationships between independent factors and the PW, QDF, ND, and BS TCMCs were individually examined (Tables 1 and 2). The results were as follows: BMI values were lowest for the participants with a QDF constitution in the biased TCMC group; participants in the ND group had the highest age, whereas their TC, TG, and Cr levels were the lowest; participants in the PW group had the highest BMI, FBG, TC, TG, and Cr levels, and this group had the highest proportion of patients with diabetes; Na and HB levels were the highest in the BS group, as were the proportions of stroke and coronary heart disease patients. The relationships between certain TCMCs and the corresponding hypertension subtypes in Western medicine are summarized in Table 4.

**Table 4:**
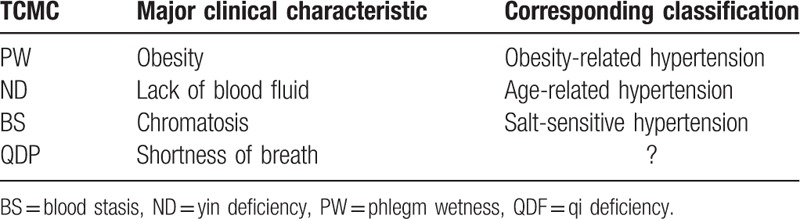
Summary of the major clinical TCMC characteristics and the possible corresponding hypertension classifications according to Western medicine criteria.

## 4 Discussion

Constitution is an ancient concept that arose approximately 2000 years ago. TCMC is a vital achievement of Chinese medicine and is used to indicate health status and susceptibility to chronic disease and to help determine treatment strategy. Therefore, TCMC has potential application in health management, disease prevention (*Zhi Wei Bing* in Chinese means preventive treatment of disease), and individual therapy. An increasing number of studies have investigated the epidemiology and influencing factors associated with TCMC in Asia, but few have incorporated extensive data including physical and blood tests as potential influencing factors. The relationship between the prevalence of chronic disease and TCMC has been investigated in small sample studies, but associations between TCMC characteristics and modern medicine have not been well summarized. To our knowledge, this study is the first to examine associations between TCMC and demographic, physical, and laboratory characteristics, and to thereby demonstrate a critical role for TCMC in the prevalence of hypertension based on extensive data. The findings of this study strengthen TCMC theory, improve our understanding of the guiding role of TCMC in the prevention of hypertension, and provide a reference for future investigations.

In total, 144,439 people were included in our present study. The participants were individuals older than 15 years from Yuelu District, Changsha, in the south-central part of China. According to The People's Government of Yuelu District of the Changsha Municipality, the total population of Yuelu District in 2015 was 880,000.^[12]^ A nationwide sampling survey showed that 17.62% of the population was younger than 15 years.^[13]^ Therefore, approximately 725,000 people older than 15 years should have been in Yuelu District; thus, approximately 20% of the residents were included in our present study. This considerable population included almost all residents who received community hospital service in Yuelu District.

The present findings show that the majority of the residents possessed the N constitution, which indicates a healthy state of innate and acquired qualities in physical appearance, physiological function, and psychological condition. The most common single-biased TCMCs were PW, QDF, and ND. The proportion of participants with the N constitution in the current study was higher than in previous studies. An investigation based on 21,948 participants in 9 provinces and cities (not including Hunan Province) showed that two-thirds of the participants had a biased TCMC and that the top 3 single-biased TCMCs were QDF, WH, and PD.^[8]^ According to Wang et al's study, the proportion of biased TCMCs differs because of geographic position. This difference may be caused by sampling-related and geographic position-related issues.

The prevalence of hypertension in our study was 16.7%, which is slightly lower than the 18.8% reported in a national general survey conducted in 2002.^[2]^ We believe this difference is acceptable because the prevalence was lower in southern China than in northern China. A highlight of the current work is that we revealed the influence of TCMC on hypertension prevalence after adjusting for confounding factors and summarized TCMC characteristics based on Western medicine theory. The PW, ND, BS, and QDF constitutions were positively associated with the prevalence of hypertension. This result is similar to that of a previous study, which demonstrated that the PW, ND, and QDF constitutions have a positive influence on hypertension.^[10]^

The PW constitution exhibited a significant association with hypertension after adjusting for confounding factors. Our results showed that participants with the PW constitution were the youngest in the biased TCMC group, but their BMI level was the highest and tended toward glucolipid metabolism problems. This finding is similar to that of a previous study, which indicated that abnormal metabolism, including hyperlipidemia, diabetes, and disability of sodium-potassium ATPase function, is involved in the development of the PW constitution.^[14]^ Therefore, hypertensive patients with the PW constitution had very similar characteristics to individuals diagnosed with obesity-associated hypertension in Western medicine. In a study by Wang et al, logistic regression analysis identified dietary habits, sleep, exercise, smoking, and body shape as the influencing factors for PW formation.^[15]^ Regrettably, we did not assess the lifestyle habits of the participants in our study; however, the relatively high number of males and the high proportion of individuals with the PW constitution who smoked and drank indicated relatively unhealthy lifestyles. Moreover, the results also suggested that lifestyle changes and attempts to prevent metabolic disorders should be considered for individuals with the PW constitution.

Approximately 4000 years ago, an ancient Chinese healer wrote in the book “The Yellow Emperor's Inner Classic • Su Wen • Debate on Yin and Yang” that “Yin is half in forty years old, then the ability of life is suppressed.” In the present study, the mean age of the participants in the ND group was the highest, and no significant abnormal physical or laboratory examinations were found in this group. Thus, the results indicate that aging is a major contributing factor to the prevalence of hypertension in patients with the ND constitution, and hypertensive patients with this constitution correspond to healthy aging people with stiffness in the aorta and arterial walls.

The Yellow Emperor's Inner Classic recorded that a high dietary intake of salt would induce a hardness of the pulse and contribute to the BS constitution. The famous Ming Dynasty medical scientist Shi-zhen Li stated that “Salt is having the smell of salt and fish as well as blood, and eating more salt will suppress circulation hemostasis and induce blood stasis.” The role of sodium is well recognized by Western medical scientists.^[16]^ Presently, there is compelling evidence connecting high-sodium diets to hypertension. The results of INTERSALT, a worldwide epidemiological study, highlight the relationship between sodium intake and hypertension at the population level.^[17]^ Interestingly, the sodium level in the present study was highest in the BS group, indicating that these individuals had a relatively high intake of salt and/or a poor ability to excrete sodium. Recently, the BS constitution was proven to play an essential role in cardiovascular disease, including coronary heart disease,^[18]^ which is consist with our results. Therefore, the initial factor promoting the development of the BS constitution is likely sodium, which means that salt-sensitive hypertension is the corresponding Western type for hypertensive patients with the BS constitution.

Another risk factor for hypertension is the QDF constitution. This constitution was reported to have a role in hypertension, particularly in females, in a previous study.^[9]^ Based on TCMC theory, the QDF constitution is characterized by a lean shape, timidity, trouble sleeping, shortness of breath, low voice, dizziness, sweating, and being prone to cold. The characteristics of the QDF constitution were not successfully summarized by the current data, and further studies are still necessary.

Hypertension involves complex interactions between genes and environmental factors, and hypertensive patients typically have unique individual clinical characteristics. Because of this variation, hypertensive patients are typically assigned to different subgroups based on biomarkers (such as renin, c-reactive protein, and homocysteine).^[19–23]^ and clinical characteristics (such as age, salt intake, obesity, and diabetes).^[24–26]^ For example, hypertensive patients with hyperhomocysteinemia have a higher risk of stroke, and folic acid supplementation is useful for prevention in such patients.^[22]^ TCMC theory is a type of empirical medicine that was developed during ancient times based on natural phenomena and has been further refined by modern medicine to explain physiological phenomena and disease.^[27,28]^ Furthermore, according to TCMC theory, biased constitution is preventable and treatable. Physical exercise, a healthy diet, and an optimistic attitude have been proven to be important protective factors for biased TCMCs. In addition, traditional Chinese herbs may be helpful for remedying biased TCMCs and certain specific diseases.^[29,30]^ One example is an antimalarial substance extracted from a plant using a low-temperature process that was discovered by the researcher Tu Youyou,^[31]^ who received the 2015 Nobel Prize in Physiology or Medicine for her work. In a word, TCMC may be a useful and simple classification system to predict the risk of hypertension and determine preventive and therapeutic effects.

There are limitations in the present study. First, we collected information from community health registry systems without random sampling. Approximately 20% of the residents in the examined geographic region were included in our present study; thus, selection bias was likely to exist. Second, some participants did not have complete clinical and laboratory data, and the proportion of patients with missing data was higher than in other studies. Third, quality control was performed by each community hospital. Extreme data points were relatively common in the present study, and sampling bias during data cleaning may have influenced the results. Finally, this was a community-based, cross-sectional study that revealed a relationship between TCMC and hypertension but without causal information. Thus, an additional cohort study is still required.

In summary, despite several limitations and limited mechanistic research, the current work showed that TCMC classification might be helpful for identifying potential health problems, preventing disease and guiding rational therapy. Better understanding of the characteristics of particular TCMCs may be useful for subtyping hypertension and could guide the evolution of precision medicine.

## 5 Conclusions

Based on TCMC theory, the health status of the residents of Yuelu District was generally good. The majority of residents were of N constitution, and the most frequent single-biased TCMCs were the PW, QDF, and ND constitutions. Moreover, the PW, ND, BS, and QDF constitutions had different characteristics and differential effects on the prevalence of hypertension; thus, TCMC may be able to help subtype hypertension.

## Supplementary Material

Supplemental Digital Content

## References

[1] Whitworth JA World Health Organization, International Society of Hypertension Writing Group. 2003 World Health Organization (WHO)/International Society of Hypertension (ISH). 2003 World Health Organization (WHO)/International Society of Hypertension (ISH). 2003 World Health Organization (WHO)/International Society of Hypertension (ISH) statement on management of hypertension. J Hypertens 2003;21:1983–92. 14597836 10.1097/00004872-200311000-00002

[2] Gu D Reynolds K Wu X Prevalence, awareness, treatment, and control of hypertension in china. Hypertension 2002;40:920–7. 12468580 10.1161/01.hyp.0000040263.94619.d5

[3] Wolf-Maier K Cooper RS Banegas JR Hypertension prevalence and blood pressure levels in 6 European countries, Canada, and the United States. JAMA 2003;289:2363–9. 12746359 10.1001/jama.289.18.2363

[4] Wang Q Constitutional Doctrine of TCM. Beijing: China Medicine Science and Technology Press; 1995.

[5] Wang Q Constitutional Doctrine of TCM. Beijing: People's Medical Publishing House; 2005.

[6] Zhu YB, Wang Q, Xiushu-Zheli. The reproducibility, reliability, and validity of the traditional Chinese Medicine constitution questionnaire. 5th Traditional Chinese Medicine constitution academic conference; 2007.

[7] China Association of Chinese Medicine. Classification and assessment of traditional Chinese Medicine constitution (ZYYXH/T157–2009). Shi Jie Zhong Xi Yi Jie He Zhi. 2009; 4:303.

[8] Wang Q Zhu Y An epidemiological traditional Chinese Medicine Constitution survey based on the 21,948 residences in 9 provinces in China. Zhong Hua Zhong Yi Yao za Zhi 2009;1:7–12.

[9] Sun Y Liu P Zhao Y Characteristics of TCM constitutions of adult Chinese women in Hong Kong and identification of related influencing factors: a cross-sectional survey. J Transl Med 2014;12:140. 24886055 10.1186/1479-5876-12-140 PMC4047264

[10] Zhu YB Wang Q Deng QW The relationship between traditional Chinese medicine constitution and hypertension. J Chin Integr Med 2010;8:40–5. 20082757

[11] Han SH Zheng JM Li KZ Influence of traditional Chinese medicine constitution type on the susceptibility of hypertensive cases to intracerebral haemorrhage. Chin J Integr Med 2014;20:923–7. 25022550 10.1007/s11655-014-1711-z

[12] The People's Government. In:. Yuelu district. Demographic statistics in Yuelu district. Available at http://www.yuelu.gov.cn/yuelugov/ylgk98/rkzk/index.html.Accessed on November 20, 2015.

[13] Statistical Bureau in Hunan Province. The bulletin of sixth population census in Hunan Province. Available at http://www.stats.gov.cn/tjsj/tjgb/rkpcgb/dfrkpcgb/201202/t20120228_30392.html. Accessed on November 20, 2015.

[14] Su Q Wang Q The characteristics of blood lipid, glucose, insulin and the activity of Na+-K+-ATP enzyme in fatty people with phlegm-wetness constitution. Zhong Yi Ji Chu Yi Xue za Zhi 1995;1:39–41.

[15] Wang Q Zhu HY Zhe LXS An investigation of the influence of phlegm-wetness constitution. Journal Beijing University of Traditional Chinese Medicine 2008;1:10–3.

[16] Cirillo M Capasso G Di Leo VA A history of salt. Am J Nephrol 1994;14:426–31. 7847480 10.1159/000168759

[17] Stamler J The INTERSALT Study: background, methods, findings, and implications. Am J Clin Nutr 1997;65 (2 suppl):626S–42S. 9022559 10.1093/ajcn/65.2.626S

[18] Zhang WT Zhe YG Hu YH The analysis of coagulation function and parameter of blood platelet in patients with coronary heart disease and blood stasis constitution. J Tradit Chin Med 2015;16:1390–3.

[19] Brown MJ Hypertension and ethnic group. BMJ 2006;332:833–6. 16601044 10.1136/bmj.332.7545.833 PMC1432176

[20] Wang TJ Gona P Larson MG Multiple biomarkers and the risk of incident hypertension. Hypertension 2007;49:432–8. 17242302 10.1161/01.HYP.0000256956.61872.aa

[21] Liu CF Gu YT Wang HY Fang NY. gamma-glutamyltransferase level and risk of hypertension: a systematic review and meta-analysis. PLoS One 2012;7:e48878. 23145005 10.1371/journal.pone.0048878 PMC3492247

[22] Huo Y Li J Qin X Efficacy of folic acid therapy in primary prevention of stroke among adults with hypertension in China: the CSPPT randomized clinical trial. JAMA 2015;313:1325–35. 25771069 10.1001/jama.2015.2274

[23] Bielecka-Dabrowa A Michalska-Kasiczak M Gluba A Biomarkers and echocardiographic predictors of myocardial dysfunction in patients with hypertension. Sci Rep 2015;5:8916. 25747153 10.1038/srep08916 PMC5390083

[24] Nagai M Ohkubo T Murakami Y Secular trends of the impact of overweight and obesity on hypertension in Japan, 1980–2010. Hypertens Res 2015;38:798. 26538013 10.1038/hr.2015.94

[25] Johnson RJ Feig DI Nakagawa T Pathogenesis of essential hypertension: historical paradigms and modern insights. J Hypertens 2008;26:381–91. 18300843 10.1097/HJH.0b013e3282f29876 PMC2742362

[26] Yokoyama H Araki S Watanabe S Prevalence of resistant hypertension and associated factors in Japanese subjects with type 2 diabetes. Diabetes Res Clin Pract 2015;110:18–25. 26361860 10.1016/j.diabres.2015.08.007

[27] Law MP Chuh AA Molinari N An investigation of the association between diet and occurrence of acne: a rational approach from a traditional Chinese medicine perspective. Clin Exp Dermatol 2010;35:31–5. 19549242 10.1111/j.1365-2230.2009.03360.x

[28] Wu SG He L Wang Q An ancient Chinese wisdom for metabolic engineering: Yin-Yang. Microb Cell Fact 2015;14:39. 25889067 10.1186/s12934-015-0219-3 PMC4374363

[29] Fleischer T Chang TT Chiang JH Integration of Chinese herbal medicine therapy improves survival of patients with chronic lymphocytic leukemia: a nationwide population-Based Cohort Study. Medicine (Baltimore) 2016;95:e3788. 27227953 10.1097/MD.0000000000003788 PMC4902377

[30] Lin SK Tsai YT Lo PC Traditional Chinese medicine therapy decreases the pneumonia risk in patients with dementia. Medicine (Baltimore) 2016;95:e4917. 27631269 10.1097/MD.0000000000004917 PMC5402612

[31] Hao C Lasker award rekindles debate over artemisinin's discovery. Available at http://news.sciencemag.org/asia/2011/09/lasker-award-rekindles-debate-over-artemisinins-discovery. Accessed on January 7, 2014.

