# Individualized prevention against hypertension based on Traditional Chinese Medicine Constitution Theory: A large community-based retrospective, STROBE-compliant study among Chinese population: Erratum

**DOI:** 10.1097/MD.0000000000010795

**Published:** 2018-05-11

In the article, “Individualized prevention against hypertension based on Traditional Chinese Medicine Constitution Theory: A large community-based retrospective, STROBE-compliant study among Chinese population”,^[1]^ which appeared in Volume 96, Issue 46 of *Medicine*, the frequency of smoking and alcohol in Table 2 were incorrect. The correct table is:

**Table 2:**
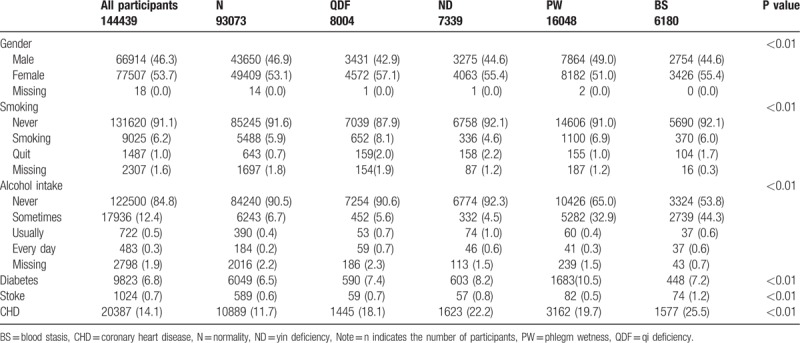
Comparison of the basic classified variables corresponding to the characteristics of different TCMCs [N (%)].
