# Supplementary material for: Individualized prevention against hypertension based on Traditional Chinese Medicine Constitution Theory: A large community-based retrospective, STROBE-compliant study among Chinese population
Source: Medicine (Baltimore). 2017 Nov 17;96(46):e8513. doi: 10.1097/MD.0000000000008513 (PMC5704799; doi:10.1097/MD.0000000000008513)
Supplement: Supplemental Digital Content [file medi-96-e8513-s001.doc]

Table S1 Questionnaires used for TCMC classification

(DOC)

**Table S1 The questionnaires of TCMCs classification**

Normality

| Please answer the following questions according to the experience and feeling in the recently one year | Never happened | A little | Sometimes | Usually | Frequently |
| --- | --- | --- | --- | --- | --- |
| （1）Do you feel physically able? | 1 | 2 | 3 | 4 | 5 |
| （2）Do you easily get tired? * | 1 | 2 | 3 | 4 | 5 |
| （3）Is your voice low and weak?* | 1 | 2 | 3 | 4 | 5 |
| （4）Do you feel chest tightness and unhappy? * | 1 | 2 | 3 | 4 | 5 |
| （5）Do you have poorer cold resistance than normal person (cold in winter, cool air condition in summer, electric fans, etc.)? * | 1 | 2 | 3 | 4 | 5 |
| （6）Can you adapt to the change of natural and social environment? | 1 | 2 | 3 | 4 | 5 |
| （7）Are you sleepless easily? * | 1 | 2 | 3 | 4 | 5 |
| （8）Do you forget easily?* | 1 | 2 | 3 | 4 | 5 |
| Note: All items marked with * is scored reversely, and transformed by formula | | | | | |

**Qi-deficiency**

| （1）Do you easily get tired? | 1 | 2 | 3 | 4 | 5 |
| --- | --- | --- | --- | --- | --- |
| （2）Are you easily short of breath (breath quickly, cannot breathe) | 1 | 2 | 3 | 4 | 5 |
| （3）Are you easily nervous? | 1 | 2 | 3 | 4 | 5 |
| （4）Are you easily faint or do you faint when stand up? | 1 | 2 | 3 | 4 | 5 |
| （5）Do you get cold easier than others? | 1 | 2 | 3 | 4 | 5 |
| （6）Do you like quiet, and are too lazy to speak? | 1 | 2 | 3 | 4 | 5 |
| （7）Is your voice low and weak? | 1 | 2 | 3 | 4 | 5 |
| （8）Are you easily abnormal sweated due to slightly activity? | 1 | 2 | 3 | 4 | 5 |

**Yang-deficiency**

| （1）Are your hands and feet cold? | 1 | 2 | 3 | 4 | 5 |
| --- | --- | --- | --- | --- | --- |
| （2）Do your stomach, back, waist and knee feel cold? * | 1 | 2 | 3 | 4 | 5 |
| （3）Do you feel cold and wear more than others? * | 1 | 2 | 3 | 4 | 5 |
| （4）Do you feel cold in winter and dislike cool air condition and electric fan? * | 1 | 2 | 3 | 4 | 5 |
| （5）Do you get cold easier than others? * | 1 | 2 | 3 | 4 | 5 |
| （6）Do you feel uncomfortable when eat (drink) cold food or feel not like eat (drink) cold food? | 1 | 2 | 3 | 4 | 5 |
| （7）Do you easily get diarrhea after eating (drinking) cold food? * | 1 | 2 | 3 | 4 | 5 |

**Yin-deficiency**

| （1）Do you feel heat for foot heart? | 1 | 2 | 3 | 4 | 5 |
| --- | --- | --- | --- | --- | --- |
| （2）Do you feel heat for body or face? | 1 | 2 | 3 | 4 | 5 |
| （3）Does your skin or mouth dry? | 1 | 2 | 3 | 4 | 5 |
| （4）Does the color of your mouth redder than others? | 1 | 2 | 3 | 4 | 5 |
| （5）Are you easily constipated or have dry faeces? | 1 | 2 | 3 | 4 | 5 |
| （6）Does your cheeks flushed or red? | 1 | 2 | 3 | 4 | 5 |
| （7）Do you feel dry eyes? | 1 | 2 | 3 | 4 | 5 |
| （8）Do you feel thirsty, and want to drink water always? | 1 | 2 | 3 | 4 | 5 |

**Phlegm-wetness**

| （1）Do you feel chest tightness or hard and full in abdomen? | 1 | 2 | 3 | 4 | 5 |
| --- | --- | --- | --- | --- | --- |
| （2）Do you feel uncomfortable or unhappy? | 1 | 2 | 3 | 4 | 5 |
| （3）Do you feel soft and full in abdomen? | 1 | 2 | 3 | 4 | 5 |
| （4）Are your forehead grease secreted? | 1 | 2 | 3 | 4 | 5 |
| （5）Does your upper eyelid more swollen than others (upper eyelid is slightly swelled) | 1 | 2 | 3 | 4 | 5 |
| （6）Do you feel sticky in the mouth? | 1 | 2 | 3 | 4 | 5 |
| （7）Do you always feel much phlegm, especially feel plugged in throat sputum? | 1 | 2 | 3 | 4 | 5 |
| （8）Do you have thick tongue or feel thick greasy tongue? | 1 | 2 | 3 | 4 | 5 |

**Wetness-heat**

| （1）Do you feel oil or sleek shine on face or nose? | 1 | 2 | 3 | 4 | 5 |
| --- | --- | --- | --- | --- | --- |
| （2）Do you easily get acne on your face or boils on your skin? | 1 | 2 | 3 | 4 | 5 |
| （3）Do you feel bit in mouth? | 1 | 2 | 3 | 4 | 5 |
| （4）Do you feel viscous and uncomfortable when defecating, with the feel of endless? | 1 | 2 | 3 | 4 | 5 |
| （5）Do you feel heat in urethra when peeing, with the dark color? | 1 | 2 | 3 | 4 | 5 |
| （6）Is your leucorrhea yellow? (limit to female) | 1 | 2 | 3 | 4 | 5 |
| （7）Is your scrotum wet? (limit to male) | 1 | 2 | 3 | 4 | 5 |

**Blood stasis**

| （1）Does your skin appear livid bruises (subcutaneous bleeding) unconsciously? | 1 | 2 | 3 | 4 | 5 |
| --- | --- | --- | --- | --- | --- |
| （2）Are there slight red in both cheeks? | 1 | 2 | 3 | 4 | 5 |
| （3）Do you hurt anywhere? | 1 | 2 | 3 | 4 | 5 |
| （4）Are your forehead grease secreted? | 1 | 2 | 3 | 4 | 5 |
| （5）Is your face dark or easy to get brown spots? | 1 | 2 | 3 | 4 | 5 |
| （6）Do you have black rim of the eye? | 1 | 2 | 3 | 4 | 5 |
| （7）Do you forget easily? | 1 | 2 | 3 | 4 | 5 |
| （8）Is the color of the mouth dark? | 1 | 2 | 3 | 4 | 5 |

**Qi-depressed**

| （1）Do you feel chest tightness and unhappy? | 1 | 2 | 3 | 4 | 5 |
| --- | --- | --- | --- | --- | --- |
| （2）Do you feel nervous and restless? | 1 | 2 | 3 | 4 | 5 |
| （3）Are you sentimental and emotionally fragile? | 1 | 2 | 3 | 4 | 5 |
| （4）Are you scared or feel afraid easily? | 1 | 2 | 3 | 4 | 5 |
| （5）Do you feel pain in rib or breast? | 1 | 2 | 3 | 4 | 5 |
| （6）Do you sign without reason? | 1 | 2 | 3 | 4 | 5 |
| （7）Do you feel foreign matter in your throat, which cannot be spitted out or swallowed? | 1 | 2 | 3 | 4 | 5 |

**Inherited special constitution**

| （1）Do you sneeze when you not get cold? | 1 | 2 | 3 | 4 | 5 |
| --- | --- | --- | --- | --- | --- |
| （2）Does your nose itch or run when you not get cold? | 1 | 2 | 3 | 4 | 5 |
| （3）Do you breathe shortly due to the change of season or geography or abnormal smell? | 1 | 2 | 3 | 4 | 5 |
| （4）Do you easily get allergy (to the drug, food, smell, dust, season change, climate change ) | 1 | 2 | 3 | 4 | 5 |
| （5）Do you get urticaria? | 1 | 2 | 3 | 4 | 5 |
| （6）Do you get purpura on your skin due to allergy? | 1 | 2 | 3 | 4 | 5 |
| （7）Does your skin turn red by a captured and appear the scratch immediately? | 1 | 2 | 3 | 4 | 5 |

Table S2 Criteria used for TCMC classification

(DOC)

**Table S2 The criteria of TCMCs classification**

| Type of the constitution | Condition | Result |
| --- | --- | --- |
| Pinghe | Score 60 | Yes |
|  | Score of other eight constitutions are all less than 30 |  |
|  | Score 60 | Basically yes |
|  | Score of at least one of the other eight constitutions are between 30~39 |  |
|  | Conditions do not meet the above descriptions | No |
| Individual Pianpo | Score 40 | Yes |
|  | Score between 30~39 | Possibly yes |
|  | Score less than 30 | No |
